# *N*-[4-Cyano-3-(trifluoro­meth­yl)phen­yl]-2-eth­oxy­benzamide

**DOI:** 10.1107/S1600536810019811

**Published:** 2010-06-05

**Authors:** S. Naveen, Basappa, H. R. Manjunath, M. A. Sridhar, J. Shashidhara Prasad, K. S. Rangappa

**Affiliations:** a Department of Physics, Sri Bhagawan Mahaveer Jain College of Engineering, Jain University, Bangalore 562 112, India; b Department of Studies in Chemistry, Manasagangotri, University of Mysore, Mysore 570 006, India; c Department of Studies in Physics, Manasagangotri, University of Mysore, Mysore 570 006, India

## Abstract

In the title compound, C_17_H_13_F_3_N_2_O_2_, the two aromatic rings are essentially coplanar, forming a dihedral angle of 2.78 (12)°. The non-H atoms of the eth­oxy group are coplanar with the attached ring [maximum deviation = 0.271 (3) Å]. An intra­molecular N—H⋯O hydrogen bond occurs. In the crystal structure, mol­ecules are linked by inter­molecular C—H⋯N and C—H⋯F hydrogen bonds.

## Related literature

For background to the biological activity of ethoxy­benzamides, see: Mantelingu *et al.* (2007 ▶). For related structures, see: Ma *et al.* (2009 ▶); Saeed *et al.* (2010 ▶). 
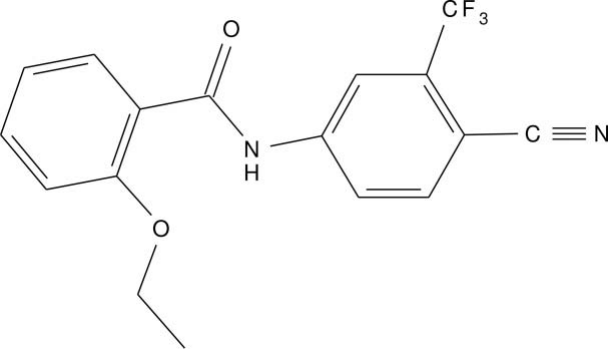


## Experimental

### 

#### Crystal data

C_17_H_13_F_3_N_2_O_2_
*M* *_r_* = 334.29
Monoclinic, 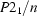

*a* = 10.5010 (13) Å
*b* = 12.8830 (16) Å
*c* = 11.6130 (14) Å
β = 101.653 (6)°
*V* = 1538.7 (3) Å^3^
*Z* = 4
Mo *K*α radiation
μ = 0.12 mm^−1^
*T* = 293 K
0.32 × 0.30 × 0.27 mm

#### Data collection

MacScience DIPLabo 32001 diffractometer
5043 measured reflections
2704 independent reflections
1896 reflections with *I* > 2σ(*I*)
*R*_int_ = 0.036

#### Refinement

*R*[*F* ^2^ > 2σ(*F* ^2^)] = 0.049
*wR*(*F* ^2^) = 0.176
*S* = 1.02
2704 reflections
217 parameters
H-atom parameters constrained
Δρ_max_ = 0.24 e Å^−3^
Δρ_min_ = −0.19 e Å^−3^

### 

Data collection: *XPRESS* (MacScience, 2002 ▶); cell refinement: *SCALEPACK* (Otwinowski & Minor, 1997 ▶); data reduction: *DENZO* (Otwinowski & Minor, 1997 ▶) and *SCALEPACK*; program(s) used to solve structure: *SHELXS97* (Sheldrick, 2008 ▶); program(s) used to refine structure: *SHELXL97* (Sheldrick, 2008 ▶); molecular graphics: *PLATON* (Spek, 2009 ▶) and *ORTEPII* (Johnson, 1976 ▶); software used to prepare material for publication: *SHELXL97*.

## Supplementary Material

Crystal structure: contains datablocks I, global. DOI: 10.1107/S1600536810019811/wn2389sup1.cif

Structure factors: contains datablocks I. DOI: 10.1107/S1600536810019811/wn2389Isup2.hkl

Additional supplementary materials:  crystallographic information; 3D view; checkCIF report

## Figures and Tables

**Table 1:** Hydrogen-bond geometry (Å, °)

*D*—H⋯*A*	*D*—H	H⋯*A*	*D*⋯*A*	*D*—H⋯*A*
N7—H1⋯O15	0.96	1.82	2.661 (2)	145
C1—H16⋯N19^i^	0.96	2.47	3.377 (3)	157
C13—H18⋯F23^ii^	0.96	2.50	3.365 (3)	150

Symmetry codes: (i) 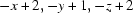; (ii) 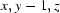.

**Fig. 1.**
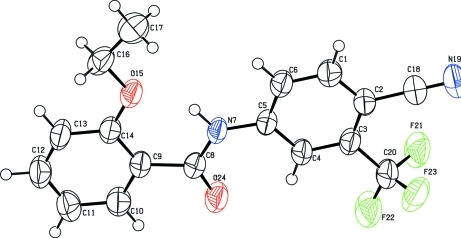
A view of the title compound, showing 50% probability displacement ellipsoids and the atom-numbering scheme. Hydrogen atoms are shown as spheres of arbitrary radius.

**Fig. 2.**
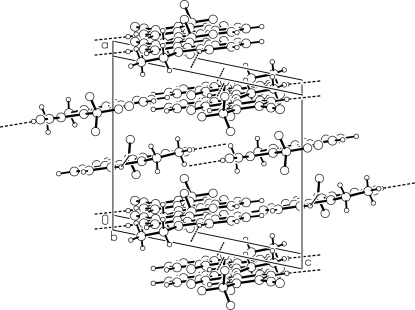
Packing diagram of the molecules, viewed down the b axis. The dashed lines represent hydrogen bonds. H atoms not involved in the hydrogen bond interactions have been omitted for clarity.

## supplementary crystallographic information

### Comment

Histone acetyl transfereses (HAT) are enzymes that acetylate conserved lysine amino acids on histone proteins by transferring the acetyl group from acetyl CoA to form ε-*N*-acetyl lysine. HAT functions to promote transcriptional activation and has significant histone acetyl transferase activity with core histones (H3 and H4), and also with nucleosome core particles. In addition, HAT inhibits cell-cycle progression and counteracts the mitogenic activity of the adenoviral oncoprotein E1A. A literature survey revealed that the small molecule KCN weakly activated the p300 histone acetyl transferase (Mantelingu *et al.* 2007). With this background, the title compound was synthesized and we report its crystal structure here.

A perspective view of the title compound is shown in Fig. 1. The bond lengths and bond angles are normal and are comparable with values reported earlier for *N*-(3,4-diethoxyphenyl)acetamide (Ma *et al.* 2009). The dihedral angle between the two aromatic rings is 2.78 (12)°, indicating that the two aromatic rings are essentially coplanar. This value differs from the value of 55.69 (3)° reported earlier (Saeed *et al.* 2010). The carbamide group connecting the two rings is *-anti-periplanar*, as indicated by the torsion angle value of -177.2 (2)° for C5—N7—C8—C9. The non-H atoms of the ethoxy group lie within the plane of the aromatic ring, as confirmed by the torsion angle value of 174.3 (2)° for C14—O15—C16—C17. In the crystal structure, the molecules exhibit both intramolecular N—H···O and intermolecular hydrogen bonds of the type C—H···N and C—H···F. The molecules exhibit layered stacking when viewed down the *b* axis, as shown in Fig. 2.

### Experimental

*N*-(4-Cyano-3-(trifluoromethyl)phenyl)-2-ethoxybenzamide was synthesized according to the procedure reported earlier (Mantelingu *et al.* 2007). The final product was obtained by crystallization using methanol as solvent. Slow evaporation of the solvent yielded colorless crystals after three days.

### Refinement

H atoms were placed at idealized positions and allowed to ride on their parent atoms with C—H and N—H distances set equal to 0.96 Å; *U*_iso_(H) = 1.2*U*_eq_(carrier atom) for all H atoms.

### Crystal data

**Table:**

C_17_H_13_F_3_N_2_O_2_	*Z* = 4
*M**_r_* = 334.29	*F*(000) = 688
Monoclinic, *P*2_1_/*n*	*D*_x_ = 1.443 Mg m^−^^3^
Hall symbol: -P 2yn	Mo *K*α radiation, λ = 0.71073 Å
*a* = 10.5010 (13) Å	µ = 0.12 mm^−^^1^
*b* = 12.8830 (16) Å	*T* = 293 K
*c* = 11.6130 (14) Å	Block, colorless
β = 101.653 (6)°	0.32 × 0.30 × 0.27 mm
*V* = 1538.7 (3) Å^3^	

### Data collection

**Table:**

MacScience DIPLabo 32001 diffractometer	1896 reflections with *I* > 2σ(*I*)
Radiation source: fine-focus sealed tube	*R*_int_ = 0.036
graphite	θ_max_ = 25.0°, θ_min_ = 2.4°
Detector resolution: 10.0 pixels mm^-1^	*h* = −12→12
ω scan	*k* = −15→15
5043 measured reflections	*l* = −13→13
2704 independent reflections	

### Refinement

**Table:**

Refinement on *F*^2^	Primary atom site location: structure-invariant direct methods
Least-squares matrix: full	Secondary atom site location: difference Fourier map
*R*[*F*^2^ > 2σ(*F*^2^)] = 0.049	Hydrogen site location: inferred from neighbouring sites
*wR*(*F*^2^) = 0.176	H-atom parameters constrained
*S* = 1.02	*w* = 1/[σ^2^(*F*_o_^2^) + (0.1179*P*)^2^] where *P* = (*F*_o_^2^ + 2*F*_c_^2^)/3
2704 reflections	(Δ/σ)_max_ = 0.001
217 parameters	Δρ_max_ = 0.24 e Å^−^^3^
0 restraints	Δρ_min_ = −0.19 e Å^−^^3^

### Special details

**Table:**

Geometry. All e.s.d.'s (except the e.s.d. in the dihedral angle between two l.s. planes) are estimated using the full covariance matrix. The cell e.s.d.'s are taken into account individually in the estimation of e.s.d.'s in distances, angles and torsion angles; correlations between e.s.d.'s in cell parameters are only used when they are defined by crystal symmetry. An approximate (isotropic) treatment of cell e.s.d.'s is used for estimating e.s.d.'s involving l.s. planes.
Refinement. Refinement of *F*^2^ against ALL reflections. The weighted *R*-factor *wR* and goodness of fit *S* are based on *F*^2^, conventional *R*-factors *R* are based on *F*, with *F* set to zero for negative *F*^2^. The threshold expression of *F*^2^ > σ(*F*^2^) is used only for calculating *R*-factors(gt) *etc*. and is not relevant to the choice of reflections for refinement. *R*-factors based on *F*^2^ are statistically about twice as large as those based on *F*, and *R*- factors based on ALL data will be even larger.

### Fractional atomic coordinates and isotropic or equivalent isotropic displacement parameters (Å^2^)

**Table:**

	*x*	*y*	*z*	*U*_iso_*/*U*_eq_	
N7	0.87506 (17)	0.12966 (13)	0.60529 (15)	0.0493 (5)	
H1	0.8991	0.0685	0.6519	0.059*	
O24	0.7968 (2)	0.17185 (12)	0.41408 (15)	0.0740 (6)	
O15	0.89269 (17)	−0.07252 (11)	0.65288 (15)	0.0610 (5)	
F21	0.75451 (17)	0.57018 (11)	0.62234 (16)	0.0844 (5)	
F22	0.79874 (19)	0.49654 (12)	0.47046 (14)	0.0996 (7)	
C6	0.9373 (2)	0.23273 (17)	0.7779 (2)	0.0566 (6)	
H6	0.9602	0.1701	0.8220	0.068*	
C14	0.8480 (2)	−0.09241 (16)	0.5358 (2)	0.0513 (6)	
C5	0.8896 (2)	0.22760 (15)	0.6581 (2)	0.0474 (5)	
C9	0.8176 (2)	−0.00775 (16)	0.45831 (19)	0.0489 (5)	
C2	0.9212 (2)	0.41873 (16)	0.7744 (2)	0.0537 (6)	
C8	0.8285 (2)	0.10574 (17)	0.4903 (2)	0.0501 (6)	
F23	0.94844 (17)	0.57372 (11)	0.59172 (17)	0.0881 (6)	
C10	0.7724 (2)	−0.0286 (2)	0.3392 (2)	0.0608 (6)	
H13	0.7506	0.0277	0.2847	0.073*	
C4	0.8589 (2)	0.31994 (16)	0.5946 (2)	0.0516 (6)	
H14	0.8274	0.3167	0.5111	0.062*	
C18	0.9345 (2)	0.51680 (19)	0.8361 (2)	0.0638 (6)	
C1	0.9528 (2)	0.32671 (18)	0.8361 (2)	0.0602 (6)	
H16	0.9846	0.3279	0.9196	0.072*	
C3	0.8748 (2)	0.41397 (16)	0.6531 (2)	0.0519 (6)	
C13	0.8323 (3)	−0.19283 (18)	0.4910 (2)	0.0645 (7)	
H18	0.8507	−0.2510	0.5433	0.077*	
N19	0.9424 (3)	0.59476 (17)	0.8833 (2)	0.0817 (7)	
C20	0.8439 (3)	0.51243 (18)	0.5837 (2)	0.0641 (7)	
C16	0.9363 (3)	−0.15806 (18)	0.7317 (2)	0.0650 (7)	
H21A	0.8635	−0.2019	0.7358	0.078*	
H21B	1.0002	−0.1970	0.7014	0.078*	
C11	0.7590 (3)	−0.1291 (2)	0.2959 (2)	0.0684 (7)	
H22	0.7290	−0.1410	0.2133	0.082*	
C12	0.7889 (3)	−0.2104 (2)	0.3733 (3)	0.0699 (7)	
H23	0.7802	−0.2804	0.3444	0.084*	
C17	0.9919 (3)	−0.1132 (2)	0.8500 (2)	0.0771 (8)	
H24A	1.0226	−0.1674	0.9054	0.092*	
H24B	0.9264	−0.0736	0.8771	0.092*	
H24C	1.0631	−0.0686	0.8426	0.092*	

### Atomic displacement parameters (Å^2^)

**Table:**

	*U*^11^	*U*^22^	*U*^33^	*U*^12^	*U*^13^	*U*^23^
N7	0.0650 (11)	0.0285 (9)	0.0529 (11)	0.0026 (8)	0.0082 (8)	0.0020 (7)
O24	0.1173 (15)	0.0391 (9)	0.0591 (11)	0.0047 (9)	0.0021 (10)	0.0044 (8)
O15	0.0839 (11)	0.0346 (8)	0.0620 (11)	0.0028 (8)	0.0085 (8)	0.0058 (7)
F21	0.0949 (11)	0.0477 (9)	0.1143 (14)	0.0226 (8)	0.0300 (10)	0.0033 (8)
F22	0.1628 (18)	0.0520 (9)	0.0731 (11)	0.0248 (10)	−0.0023 (10)	0.0071 (8)
C6	0.0748 (15)	0.0384 (12)	0.0545 (14)	0.0010 (11)	0.0080 (11)	0.0026 (10)
C14	0.0574 (13)	0.0365 (11)	0.0605 (14)	−0.0025 (9)	0.0134 (10)	−0.0019 (9)
C5	0.0554 (12)	0.0329 (10)	0.0547 (13)	−0.0006 (9)	0.0129 (9)	−0.0007 (9)
C9	0.0533 (12)	0.0353 (11)	0.0585 (14)	0.0004 (9)	0.0118 (10)	−0.0014 (10)
C2	0.0624 (13)	0.0408 (12)	0.0594 (15)	−0.0033 (10)	0.0159 (11)	−0.0071 (10)
C8	0.0585 (13)	0.0361 (11)	0.0557 (14)	0.0011 (9)	0.0117 (10)	0.0022 (10)
F23	0.1009 (12)	0.0474 (8)	0.1229 (15)	−0.0056 (8)	0.0386 (10)	0.0167 (8)
C10	0.0701 (15)	0.0484 (13)	0.0618 (15)	−0.0015 (11)	0.0080 (11)	−0.0035 (11)
C4	0.0682 (14)	0.0342 (11)	0.0521 (13)	0.0048 (10)	0.0116 (10)	−0.0004 (9)
C18	0.0779 (16)	0.0476 (14)	0.0669 (15)	−0.0019 (12)	0.0174 (12)	−0.0099 (12)
C1	0.0761 (16)	0.0492 (13)	0.0534 (13)	−0.0027 (11)	0.0084 (11)	−0.0021 (11)
C3	0.0587 (13)	0.0338 (11)	0.0657 (15)	0.0021 (9)	0.0187 (11)	0.0008 (10)
C13	0.0798 (16)	0.0325 (11)	0.0818 (18)	−0.0040 (11)	0.0178 (13)	−0.0010 (11)
N19	0.110 (2)	0.0529 (13)	0.0824 (17)	−0.0022 (12)	0.0193 (14)	−0.0211 (12)
C20	0.0848 (17)	0.0377 (12)	0.0706 (17)	0.0092 (12)	0.0173 (13)	−0.0013 (11)
C16	0.0742 (15)	0.0465 (13)	0.0750 (17)	0.0069 (12)	0.0167 (13)	0.0178 (12)
C11	0.0798 (17)	0.0554 (15)	0.0681 (16)	−0.0080 (13)	0.0105 (13)	−0.0169 (13)
C12	0.0812 (17)	0.0438 (13)	0.086 (2)	−0.0117 (12)	0.0192 (14)	−0.0166 (13)
C17	0.0840 (18)	0.0718 (18)	0.0721 (18)	0.0127 (15)	0.0080 (14)	0.0129 (14)

### Geometric parameters (Å, °)

**Table:**

N7—C8	1.362 (3)	F23—C20	1.340 (3)
N7—C5	1.398 (3)	C10—C11	1.386 (3)
N7—H1	0.9600	C10—H13	0.9600
O24—C8	1.225 (3)	C4—C3	1.382 (3)
O15—C14	1.370 (3)	C4—H14	0.9598
O15—C16	1.447 (3)	C18—N19	1.139 (3)
F21—C20	1.344 (3)	C1—H16	0.9599
F22—C20	1.321 (3)	C3—C20	1.503 (3)
C6—C1	1.380 (3)	C13—C12	1.370 (4)
C6—C5	1.381 (3)	C13—H18	0.9600
C6—H6	0.9600	C16—C17	1.496 (4)
C14—C13	1.392 (3)	C16—H21A	0.9600
C14—C9	1.409 (3)	C16—H21B	0.9599
C5—C4	1.403 (3)	C11—C12	1.374 (4)
C9—C10	1.395 (3)	C11—H22	0.9600
C9—C8	1.507 (3)	C12—H23	0.9601
C2—C1	1.390 (3)	C17—H24A	0.9600
C2—C3	1.395 (3)	C17—H24B	0.9600
C2—C18	1.445 (3)	C17—H24C	0.9600
			
C8—N7—C5	128.36 (18)	C2—C1—H16	120.3
C8—N7—H1	111.7	C4—C3—C2	121.2 (2)
C5—N7—H1	120.0	C4—C3—C20	119.0 (2)
C14—O15—C16	119.14 (18)	C2—C3—C20	119.9 (2)
C1—C6—C5	121.2 (2)	C12—C13—C14	121.1 (2)
C1—C6—H6	118.9	C12—C13—H18	119.2
C5—C6—H6	119.9	C14—C13—H18	119.7
O15—C14—C13	122.4 (2)	F22—C20—F23	106.6 (2)
O15—C14—C9	118.49 (19)	F22—C20—F21	106.5 (2)
C13—C14—C9	119.1 (2)	F23—C20—F21	105.6 (2)
C6—C5—N7	118.07 (19)	F22—C20—C3	113.5 (2)
C6—C5—C4	119.1 (2)	F23—C20—C3	112.1 (2)
N7—C5—C4	122.8 (2)	F21—C20—C3	112.2 (2)
C10—C9—C14	118.1 (2)	O15—C16—C17	107.6 (2)
C10—C9—C8	115.14 (19)	O15—C16—H21A	109.1
C14—C9—C8	126.7 (2)	C17—C16—H21A	110.4
C1—C2—C3	118.8 (2)	O15—C16—H21B	108.7
C1—C2—C18	120.0 (2)	C17—C16—H21B	111.5
C3—C2—C18	121.2 (2)	H21A—C16—H21B	109.5
O24—C8—N7	122.9 (2)	C12—C11—C10	118.8 (2)
O24—C8—C9	120.1 (2)	C12—C11—H22	121.2
N7—C8—C9	117.08 (18)	C10—C11—H22	120.1
C11—C10—C9	122.0 (2)	C13—C12—C11	120.9 (2)
C11—C10—H13	118.2	C13—C12—H23	119.5
C9—C10—H13	119.8	C11—C12—H23	119.7
C3—C4—C5	119.5 (2)	C16—C17—H24A	110.5
C3—C4—H14	121.1	C16—C17—H24B	109.5
C5—C4—H14	119.4	H24A—C17—H24B	109.5
N19—C18—C2	178.5 (3)	C16—C17—H24C	108.4
C6—C1—C2	120.2 (2)	H24A—C17—H24C	109.5
C6—C1—H16	119.4	H24B—C17—H24C	109.5

### Hydrogen-bond geometry (Å, °)

**Table:**

*D*—H···*A*	*D*—H	H···*A*	*D*···*A*	*D*—H···*A*
N7—H1···O15i	0.96	1.82	2.661 (2)	145
C1—H16···N19^i^	0.96	2.47	3.377 (3)	157
C13—H18···F23^ii^	0.96	2.50	3.365 (3)	150

Symmetry codes: i; (i) −*x*+2, −*y*+1, −*z*+2; (ii) *x*, *y*−1, *z*.
